# The genome sequence of the common adder, *Vipera berus *(Linnaeus, 1758)

**DOI:** 10.12688/wellcomeopenres.23470.2

**Published:** 2025-09-26

**Authors:** John Benjamin (Ben) Owens, Wolfgang Wüster, John Mulley, Stuart Graham, Rhys Morgan, Axel Barlow, Wellcome Sanger Institute Tree of Life Management, Samples and Laboratory team, Wellcome Sanger Institute Scientific Operations: Sequencing Operations, Wellcome Sanger Institute Tree of Life Core Informatics team, Tree of Life Core Informatics collective, Darwin Tree of Life Consortium

**Affiliations:** 1 Molecular Ecology and Evolution at Bangor (MEEB), School of Natural Sciences, Bangor University, Environment Centre Wales, Bangor, Wales, LL57 2UW, UK; 2 Captive & Field Herpetology Ltd, Holyhead, Anglesey, Wales, LL65 1YU, UK; 3 School of Natural Sciences, Bangor University, Bangor, Wales, UK

**Keywords:** Vipera berus, common adder, genome sequence, chromosomal, Squamata

## Abstract

We present a genome assembly from an individual female *Vipera berus* (common adder; Chordata; Lepidosauria; Squamata; Viperidae). The haplotype-resolved assembly contains two haplotypes with total lengths of 1,695.0 megabases and 1,476.7 megabases, respectively. Most of haplotype 1 (98.45%) is scaffolded into 19 chromosomal pseudomolecules, while haplotype 2 is assembled to scaffold level. Haplotype 1 achieves the Earth Biogenome Project reference standard of 6.C.Q52. The mitochondrial genome has also been assembled and is 17.35 kilobases in length.

## Species taxonomy

Eukaryota; Opisthokonta; Metazoa; Eumetazoa; Bilateria; Deuterostomia; Chordata; Craniata; Vertebrata; Gnathostomata; Teleostomi; Euteleostomi; Sarcopterygii; Dipnotetrapodomorpha; Tetrapoda; Amniota; Sauropsida; Sauria; Lepidosauria; Squamata; Bifurcata; Unidentata; Episquamata; Toxicofera; Serpentes; Colubroidea; Viperidae; Viperinae; *Vipera*; *Vipera berus* (Linnaeus, 1758) (NCBI:txid)

## Background

The common or European adder ( *Vipera berus*) is a small, viviparous and geographically widespread species of viper in the family Viperidae. It ranges from the United Kingdom, where it is the only venomous snake species, across Eurasia to the Pacific Ocean ( Munkhbayar *et al.*, 2021). The adder has the largest geographic distribution of any snake species, and is the only snake to occur north of the Arctic Circle in Scandinavia. Several subspecies ( *V. b. barani, V. b. bosniensis, V. b. marasso, V. b. sachalinensis*, *V. b. walser*) are recognised along the southern edges of its distribution, but the great majority of the range is occupied by the nominate form, *V. b. berus* ( Dufresnes *et al.*, 2024). The species typically attains an adult length of approximately 50–60 cm, is relatively thick bodied and has a distinctive black, zig-zag dorsal patten. Females are generally larger and often browner in colouration whereas males are often more greyish or even silver. Melanistic specimens can be common in many parts of its range. Adders are active between March and September and hibernate during the winter. Their diet consists of a range of vertebrates including small mammals, lizards, anurans and small birds ( Andrén, 1982; Luiselli *et al.*, 1995).

Adder bites are uncommon with an estimated 50 to 100 bites occurring annually in the UK ( Coulson *et al.*, 2013) and as few as 14 deaths in the last 150 years ( Jackson *et al.*, 2016).

The species occupies a wide range of habitats across its range, but tends to have specific requirements within any one region. In the UK, it is found in habitats such as heathlands, grasslands, sand dune systems, forest edges and rocky montane slopes. This habitat specificity, coupled with a low dispersal capability, results in an inability to cross habitats such as agricultural areas, rendering it vulnerable to habitat fragmentation ( Phelps, 2004).

Although *V. berus* is classed by the IUCN as a Least Concern species globally ( Munkhbayar *et al.*, 2021), the species is suffering rapid population declines in many parts of its range. In the United Kingdom most populations in England are thought to be at risk of extinction within the next decade ( Gardner *et al.*, 2019), and it is listed as threatened in multiple European national Red Lists, e.g., VU in Germany, France and England, EN in Switzerland ( Foster *et al.*, 2021; Rote-Liste-Gremium Amphibien und Reptilien, 2020; UICN France *et al.*, 2015; Ursenbacher *et al.*, 2009). The decline is thought to be primarily the result of severe habitat degradation and fragmentation, resulting in dwindling population sizes and inbreeding ( Ball *et al.*, 2020; Pozzi *et al.*, 2023), although climate change looms as a further threat ( Madsen *et al.*, 2023).

The adder has become a model system in the study of reproductive strategies ( Ursenbacher *et al.*, 2009), pattern polymorphism ( Madsen *et al.*, 2022), conservation genetics and genetic rescue ( Madsen *et al.*, 1999; Madsen *et al.*, 2020) and the likely impact of climate change on reptiles at higher latitudes ( Dezetter *et al.*, 2021; Madsen *et al.*, 2023). Moreover, the species shows considerable variation in venom composition across its vast range ( Di Nicola *et al.*, 2021; Malina *et al.*, 2017). This first high-quality, chromosome-level adder reference genome will be of value to biologists developing studies of this model system, and particularly for those seeking to understand the underlying genetic background of population declines across anthropogenically altered landscapes, devising reintroduction or genetic rescue strategies or seeking to predict and understand potential adaptive responses to climate change.

## Genome sequence report

**Figure 1.**
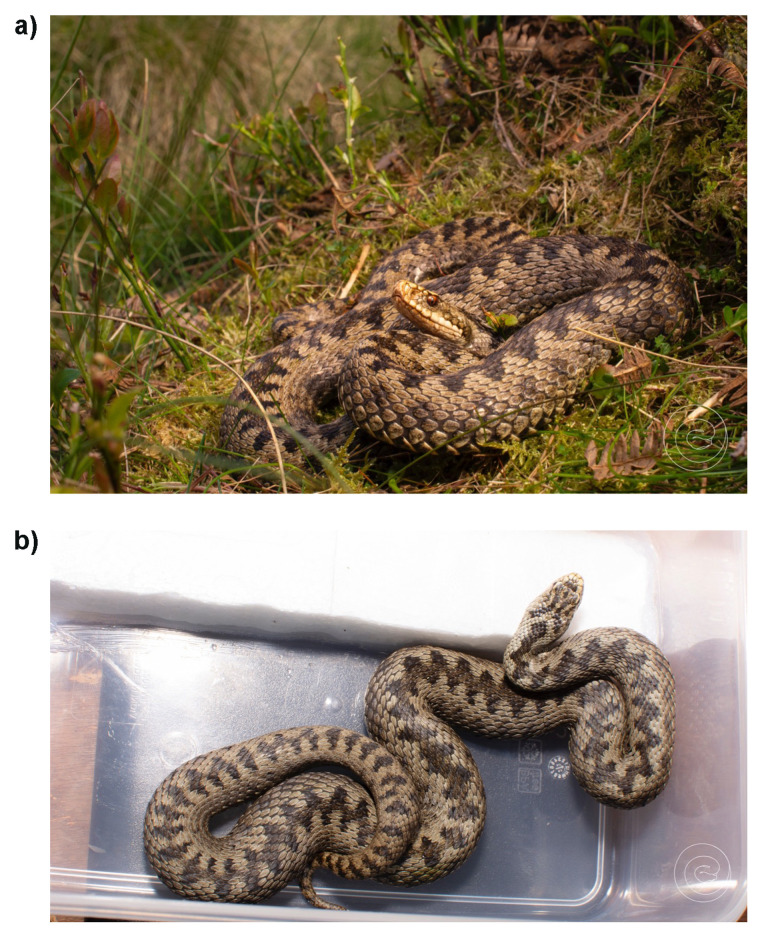
**a**) The female adder captured for blood sampling for a reference genome for the Darwin Tree of Life (DToL) project. Photograph was taken approximately ten minutes following release as the individual returned to the site of capture to bask. Cannock Chase, England. Photograph by John Benjamin (Ben) Owens. **b**) Dorsal photo of the female adder prior to release. Note that the head and dorsal pattern may be useful in the future for identifying the same individual. Photograph by John Benjamin (Ben) Owens.

The genome of *Vipera berus* ( Figure 1) was sequenced using Pacific Biosciences single-molecule HiFi long reads, generating a total of 187.72 Gb (gigabases) from 15.49 million reads, providing an estimated 61-fold coverage. Primary assembly contigs were scaffolded with chromosome conformation Hi-C data, which produced 300.91 Gb from 1,992.81 million reads. Specimen and sequencing details are summarised in Table 1.

**Table 1.** Specimen and sequencing data for *Vipera berus*.

Project information			
**Study title**	Vipera berus (adder)		
**Umbrella BioProject**	PRJEB73681		
**Species**	*Vipera berus*		
**BioSample**	SAMEA114293681		
**NCBI taxonomy ID**	31155		
Specimen information			
**Technology**	**ToLID**	**BioSample accession**	**Organism part**
**PacBio long read sequencing**	rVipBer3	SAMEA114293682	blood
**Hi-C sequencing**	rVipBer3	SAMEA114293682	blood
**RNA sequencing**	rVipBer3	SAMEA114293682	blood
Sequencing information			
**Platform**	**Run accession**	**Read count**	**Base count** **(Gb)**
**Illumina NovaSeq X (Hi-C)**	ERR12743665	1.99e+09	300.91
**Revio (PacBio)**	ERR12736907	6.72e+06	92.62
**Revio (PacBio)**	ERR12736908	8.77e+06	95.11
**Illumina NovaSeq X (RNA)**	ERR13093653	1.11e+08	16.75

The two haplotypes were combined for curation. Manual assembly curation corrected 133 missing joins or mis-joins. This increased the assembly length by 1.85% and reduced the scaffold number by 3.82%.

**Figure 2.**
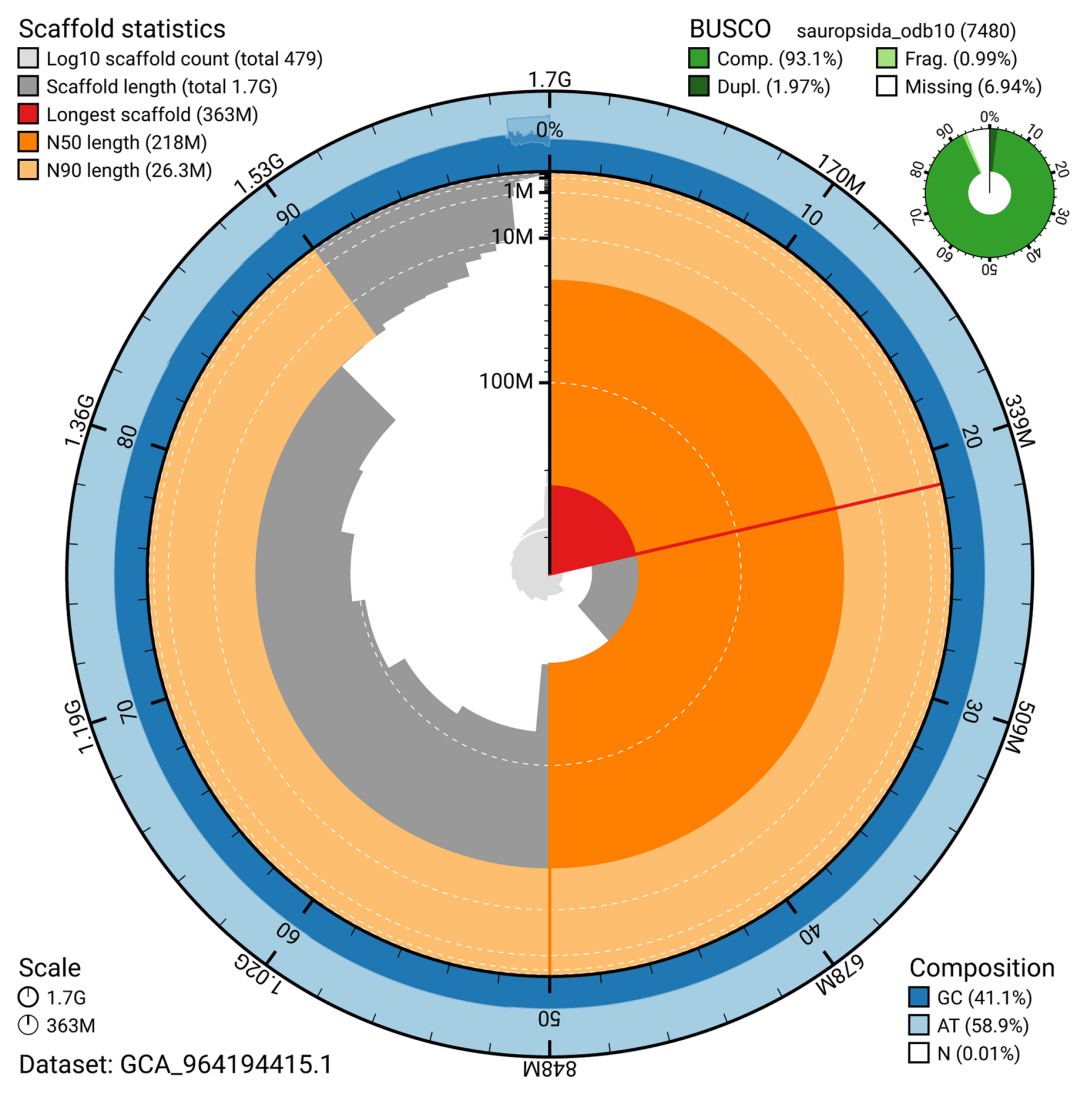
Genome assembly of *Vipera berus*, rVipBer3.hap1.1: metrics. The BlobToolKit snail plot provides an overview of assembly metrics and BUSCO gene completeness. The circumference represents the length of the whole genome sequence, and the main plot is divided into 1,000 bins around the circumference. The outermost blue tracks display the distribution of GC, AT, and N percentages across the bins. Scaffolds are arranged clockwise from longest to shortest and are depicted in dark grey. The longest scaffold is indicated by the red arc, and the deeper orange and pale orange arcs represent the N50 and N90 lengths. A light grey spiral at the centre shows the cumulative scaffold count on a logarithmic scale. A summary of complete, fragmented, duplicated, and missing BUSCO genes in the sauropsids_odb10 set is presented at the top right. An interactive version of this figure is available at https://blobtoolkit.genomehubs.org/view/Vipera_berus/dataset/GCA_964194415.1/snail.

**Figure 3.**
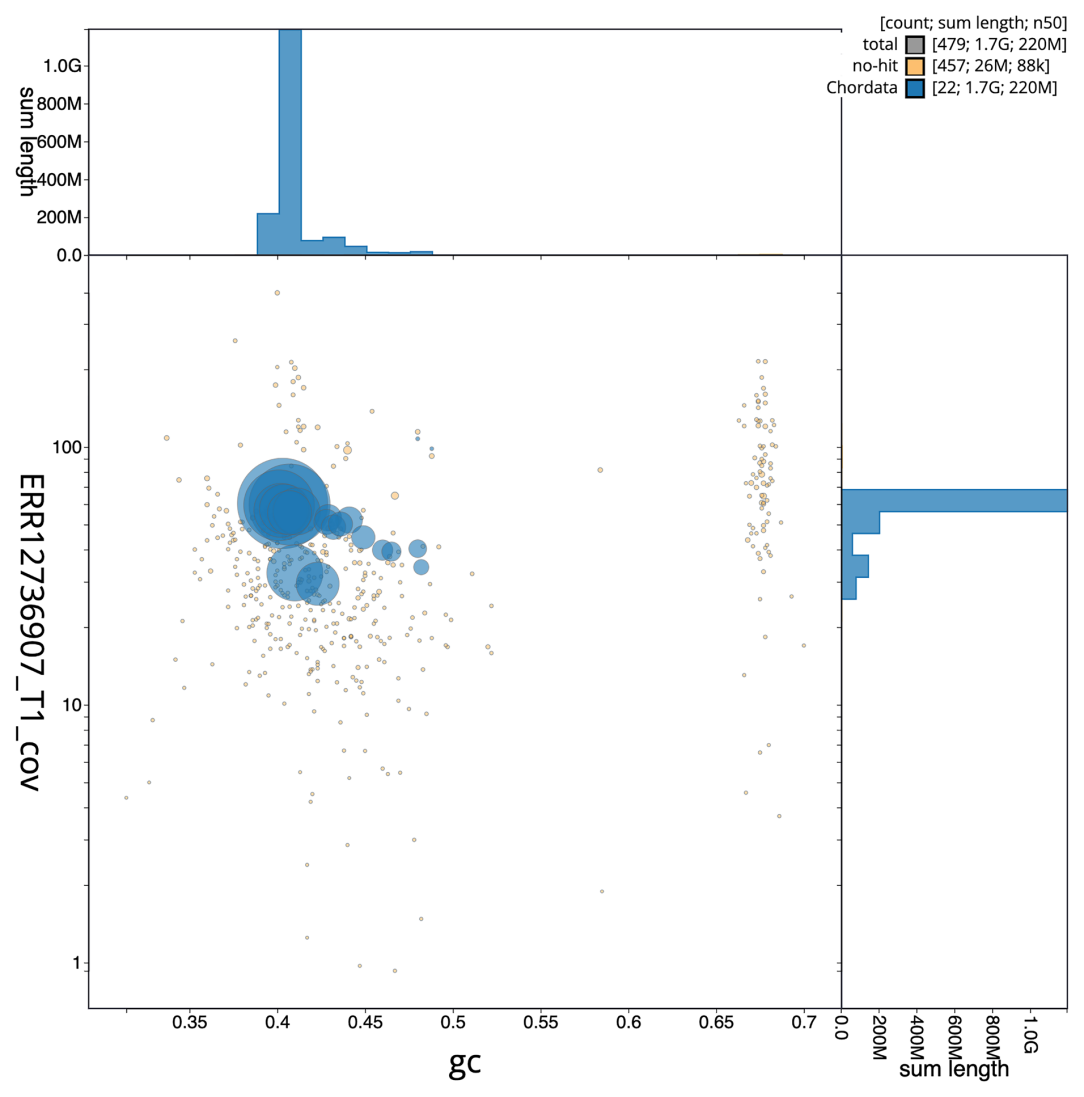
Genome assembly of *Vipera berus*, rVipBer3.hap1.1: BlobToolKit GC-coverage plot. Blob plot showing sequence coverage (vertical axis) and GC content (horizontal axis). The circles represent scaffolds, with the size proportional to scaffold length and the colour representing phylum membership. The histograms along the axes display the total length of sequences distributed across different levels of coverage and GC content. An interactive version of this figure is available at https://blobtoolkit.genomehubs.org/view/Vipera_berus/dataset/GCA_964194415.1/blob.

**Figure 4.**
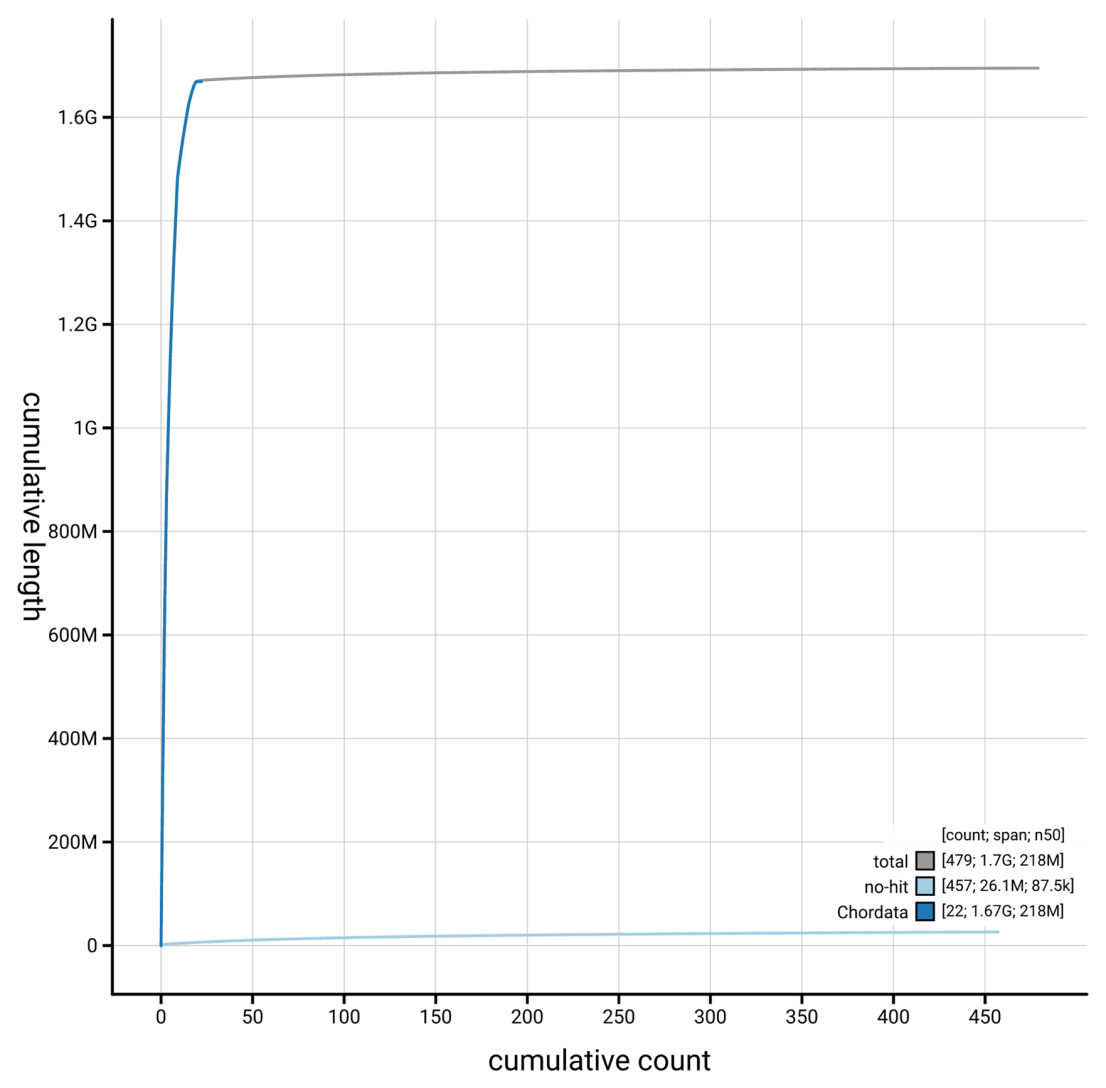
Genome assembly of *Vipera berus* rVipBer3.hap1.1: BlobToolKit cumulative sequence plot. The grey line shows cumulative length for all scaffolds. Coloured lines show cumulative lengths of scaffolds assigned to each phylum using the buscogenes taxrule. An interactive version of this figure is available at https://blobtoolkit.genomehubs.org/view/Vipera_berus/dataset/GCA_964194415.1/cumulative.

The final haplotype 1 assembly has a total length of 1,695.00 Mb in 478 sequence scaffolds, with 1,057 gaps, and a scaffold N50 of 218.4 Mb ( Table 2). The snail plot in Figure 2 provides a summary of the assembly statistics, while the distribution of assembly scaffolds on GC proportion and coverage is shown in Figure 3. The cumulative assembly plot in Figure 4 shows curves for subsets of scaffolds assigned to different phyla.

**Table 2.** Genome assembly data for the *Vipera berus* assembly.

Genome assembly	Haplotype 1	Haplotype 2
Assembly name	rVipBer3.hap1.1	rVipBer3.hap2.1
Assembly accession	GCA_964194415.1	GCA_964194405.1
Assembly level	chromosome	scaffold
Span (Mb)	1,695.0	1,476.7
Number of contigs	1,535	1,256
Number of scaffolds	478	348
Assembly metrics *	**Haplotype 1**	**Haplotype 2**
Contig N50 length (≥ 1 Mb)	4.69 Mb	4.76 Mb
Scaffold N50 length (= chromosome N50)	218.4 Mb	216.58 Mb
Consensus quality (QV) (≥ 40)	52.1	51.9
*k*-mer completeness	93.12%	84.69%
*k*-mer completeness combined haplotypes (≥ 95%)	99.09%	
BUSCO ** (S > 90%; D < 5%)	C:93.0%[S:91.0%,D:2.0%], F:1.1%,M:5.9%,n:7,480	C:88.0%[S:86.6%,D:1.4%], F:1.1%,M:10.9%,n:7,480
Percentage of assembly mapped to chromosomes (≥ 90%)	98.45%	-
Sex chromosomes (localised homologous pairs)	ZW	-
Organelles (one complete allele)	Mitochondrial genome: 17.35 kb	-

* Assembly metric benchmarks are adapted from Rhie *et al.* (2021) and the Earth BioGenome Project Report on Assembly Standards September 2024. ** BUSCO scores based on the BUSCO set using version 5.4.3. C = complete [S = single copy, D = duplicated], F = fragmented, M = missing, n = number of orthologues in comparison.

**Figure 5.**
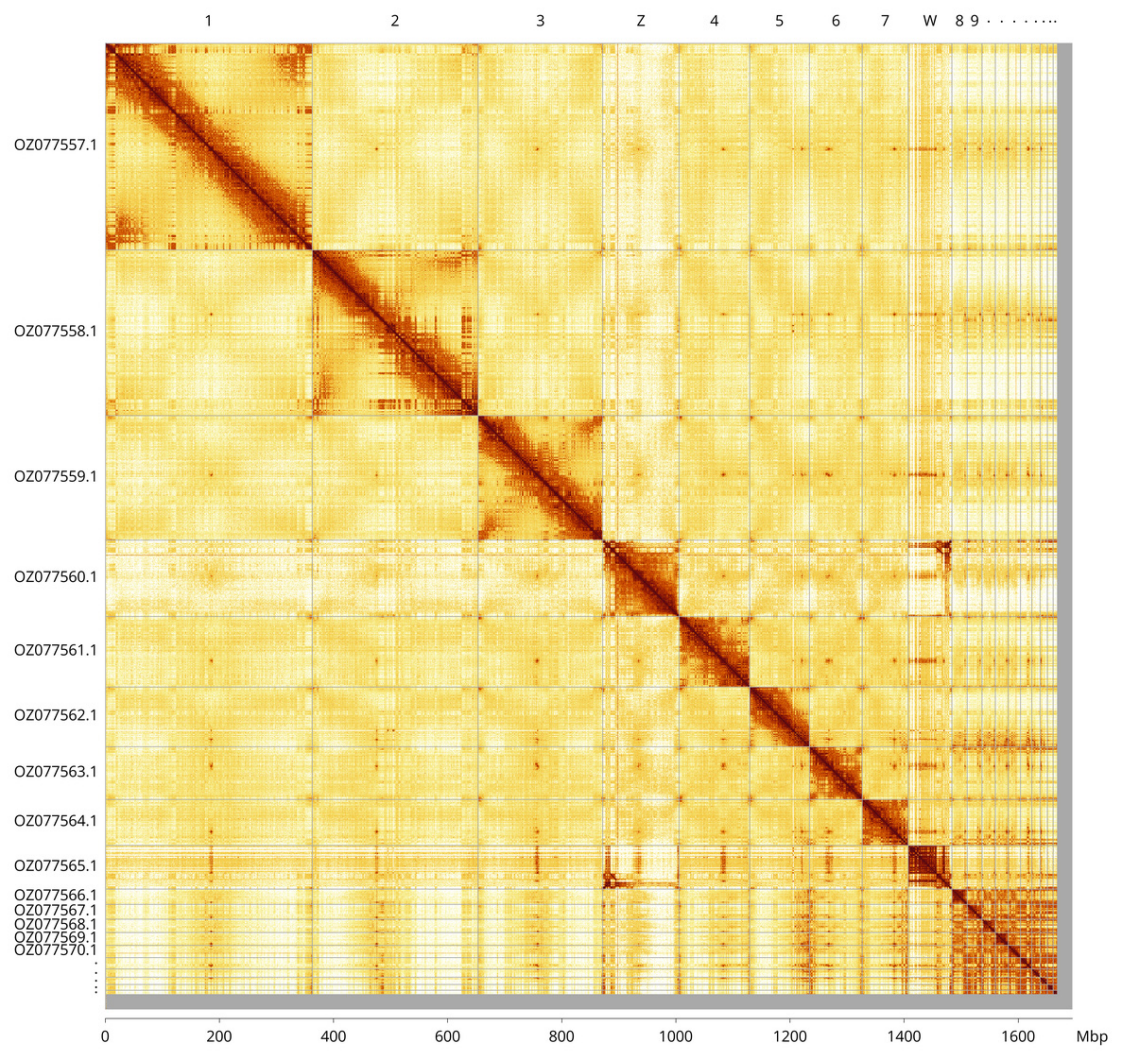
Genome assembly of *Vipera berus* rVipBer3.hap1.1: Hi-C contact map of the rVipBer3.hap1.1 assembly, visualised using PretextView and PretextSnapshot. Chromosomes are shown in order of size from left to right and top to bottom. An interactive version of this figure in HiGlass may be viewed at https://genome-note-higlass.tol.sanger.ac.uk/l/?d=BZ0QBuloRl62Y9iTrYZq1g.

Most (98.45%) of the assembly sequence was assigned to 19 chromosomal-level scaffolds, representing 17 autosomes and the Z and W sex chromosomes. Chromosome-scale scaffolds confirmed by the Hi-C data are named in order of size ( Figure 5; Table 3). Chromosomes Z and W were assigned based on the PacBio read coverage statistics and Hi-C signal.

**Table 3.** Chromosomal pseudomolecules in the genome assembly of *Vipera berus*, rVipBer3.

INSDC accession	Name	Length (Mb)	GC%
OZ077557.1	1	362.93	40.5
OZ077558.1	2	290.63	40.5
OZ077559.1	3	218.4	40
OZ077561.1	4	123.51	40
OZ077562.1	5	104.75	40.5
OZ077563.1	6	92.56	41
OZ077564.1	7	80.61	40.5
OZ077566.1	8	26.57	43
OZ077567.1	9	26.26	44
OZ077568.1	10	23.42	43
OZ077569.1	11	22.19	43
OZ077570.1	12	21.63	43.5
OZ077571.1	13	19.92	45
OZ077572.1	14	14.45	46
OZ077573.1	15	12.63	46.5
OZ077574.1	16	10.45	48
OZ077575.1	17	7.21	48
OZ077565.1	W	76.64	42.5
OZ077560.1	Z	134.0	41
OZ077576.1	MT	0.02	40.5

The mitochondrial genome was also assembled and is included both as a contig within the multifasta file of the genome submission and as a standalone record in GenBank.

The estimated Quality Value (QV) of the final haplotype 1 assembly is 52.1 with *k*-mer completeness of 93.12%, and the assembly has a BUSCO v5.4.3 completeness of 93.0% (single = 91.0%, duplicated = 2.0%), using the sauropsida_odb10 reference set ( *n* = 7,480).

For haplotype 2, the estimated Quality Value (QV) of the final assembly is 51.9 with *k*-mer completeness of 84.69%. The assembly has a BUSCO v5.4.3 completeness of 88.0% (single = 86.6%, duplicated = 1.4%), using the sauropsida_odb10 reference set ( *n* = 7,480). The *k*-mer completeness for the combined assemblies was 99.09%.

Table 1 lists the assembly metric benchmarks adapted from Rhie *et al.* (2021) and the Earth BioGenome Project Report on Assembly Standards September 2024. The EBP metric, calculated for the haplotype 1, is **6.C.Q52**, meeting the recommended reference standard.

Metadata for specimens, BOLD barcode results, spectra estimates, sequencing runs, contaminants and pre-curation assembly statistics are given at https://links.tol.sanger.ac.uk/species/31155.

## Methods

### Sample acquisition

An adult female adder was collected from Cannock Chase, England (specimen ID SAN00002972, ToLID rVipBer3) on 2023-05-14. The individual was transported to Bangor University, Wales, where a blood sample was collected intravenously from the tail in accordance with the methodology of the home office licence (PPL number PC44793B6). The blood sample was snap-frozen at –80 °C and stored at this temperature until transport to the Wellcome Sanger Institute, Hinxton. The individual was released on 2023-05-15 at the exact point of capture, under similar climatic conditions and the same time of day. The authors note that the individual was unharmed and observed basking within ten minutes of being released.

### Nucleic acid extraction

The workflow for high molecular weight (HMW) DNA extraction at the Wellcome Sanger Institute (WSI) Tree of Life Core Laboratory includes a sequence of core procedures: sample preparation and homogenisation, DNA extraction, fragmentation and purification. Detailed protocols are available on protocols.io ( Denton *et al.*, 2023c). The rVipBer3 sample was prepared for DNA extraction by weighing and dissecting it on dry ice ( Jay *et al.*, 2023). The blood sample was homogenised using a PowerMasher II tissue disruptor ( Denton *et al.*, 2023a). HMW DNA was extracted using the Manual Nucleated Blood Nanobind protocol ( Denton *et al.*, 2023b). DNA was sheared into an average fragment size of 12–20 kb in a Megaruptor 3 system ( Bates *et al.*, 2023). Sheared DNA was purified by solid-phase reversible immobilisation, using AMPure PB beads to eliminate shorter fragments and concentrate the DNA ( Oatley *et al.*, 2023). The concentration of the sheared and purified DNA was assessed using a Nanodrop spectrophotometer and Qubit Fluorometer using the Qubit dsDNA High Sensitivity Assay kit. Fragment size distribution was evaluated by running the sample on the FemtoPulse system.

RNA was extracted from blood tissue of rVipBer3 in the Tree of Life Laboratory at the WSI using the RNA Extraction: Automated MagMax™ *mir*Vana protocol ( do Amaral *et al.*, 2023). The RNA concentration was assessed using a Nanodrop spectrophotometer and a Qubit Fluorometer using the Qubit RNA Broad-Range Assay kit. Analysis of the integrity of the RNA was done using the Agilent RNA 6000 Pico Kit and Eukaryotic Total RNA assay.

### Hi-C preparation

Tissue from the blood of the rVipBer3 sample was processed at the WSI Scientific Operations core, using the Arima-HiC v2 kit. Frozen tissue (stored at –80 °C) was fixed, and the DNA crosslinked using a TC buffer with 22% formaldehyde. After crosslinking, the tissue was homogenised using the Diagnocine Power Masher-II and BioMasher-II tubes and pestles. Following the kit manufacturer's instructions, crosslinked DNA was digested using a restriction enzyme master mix. The 5’-overhangs were then filled in and labelled with biotinylated nucleotides and proximally ligated. An overnight incubation was carried out for enzymes to digest remaining proteins and for crosslinks to reverse. A clean up was performed with SPRIselect beads prior to library preparation.

### Library preparation and sequencing

Pacific Biosciences SMRTbell libraries were constructed using the Revio HiFi prep kit, according to the manufacturers’ instructions. DNA sequencing was performed by the Scientific Operations core at the WSI on a Pacific Biosciences Revio instrument.

For Hi-C library preparation, DNA was fragmented to a size of 400 to 600 bp using a Covaris E220 sonicator. The DNA was then enriched, barcoded, and amplified using the NEBNext Ultra II DNA Library Prep Kit following manufacturers’ instructions. The Hi-C sequencing was performed using paired-end sequencing with a read length of 150 bp on an Illumina NovaSeq X instrument.

Poly(A) RNA-Seq libraries were constructed using the NEB Ultra II RNA Library Prep kit, following the manufacturer’s instructions. RNA sequencing was performed on the Illumina NovaSeq X instrument.

### Genome assembly, curation and evaluation

***Assembly***

The HiFi reads were first assembled using Hifiasm ( Cheng *et al.*, 2021; Cheng *et al.*, 2022) in Hi-C phasing mode, resulting in a pair of haplotype-resolved assemblies. The Hi-C reads were mapped to the primary contigs using bwa-mem2 ( Vasimuddin *et al.*, 2019). The contigs were further scaffolded using the provided Hi-C data ( Rao *et al.*, 2014) in YaHS ( Zhou *et al.*, 2023) using the --break option for handling potential misassemblies. The scaffolded assemblies were evaluated using Gfastats ( Formenti *et al.*, 2022), BUSCO ( Manni *et al.*, 2021) and MERQURY.FK ( Rhie *et al.*, 2020).

The mitochondrial genome was assembled using MitoHiFi ( Uliano-Silva *et al.*, 2023), which runs MitoFinder ( Allio *et al.*, 2020) and uses these annotations to select the final mitochondrial contig and to ensure the general quality of the sequence.

***Assembly curation***

The assembly was decontaminated using the Assembly Screen for Cobionts and Contaminants (ASCC) pipeline (article in preparation). Flat files and maps used in curation were generated in TreeVal ( Pointon *et al.*, 2023). Manual curation was primarily conducted using PretextView ( Harry, 2022), with additional insights provided by JBrowse2 ( Diesh *et al.*, 2023) and HiGlass ( Kerpedjiev *et al.*, 2018). Scaffolds were visually inspected and corrected as described by Howe *et al*. (2021). Any identified contamination, missed joins, and mis-joins were corrected, and duplicate sequences were tagged and removed. The sex chromosomes were identified based on read coverage statistics. The curation process is documented at https://gitlab.com/wtsi-grit/rapid-curation (article in preparation).

***Evaluation of the final assembly***

The readmapping pipeline aligns the Hi-C reads using bwa-mem2 ( Vasimuddin *et al.*, 2019) and combines the alignment files with SAMtools ( Danecek *et al.*, 2021). The genomenote pipeline converts the Hi-C alignments into a contact map using BEDTools ( Quinlan & Hall, 2010) and the Cooler tool suite ( Abdennur & Mirny, 2020). The contact map is visualised in HiGlass ( Kerpedjiev *et al.*, 2018). This pipeline also computes *k*-mer completeness and QV consensus quality values with FastK and MERQURY.FK, and runs BUSCO ( Manni *et al.*, 2021) to assess completeness.

The blobtoolkit ( Muffato *et al.*, 2024) pipeline is a Nextflow ( Di Tommaso *et al.*, 2017) port of the previous Snakemake Blobtoolkit pipeline ( Challis *et al.*, 2020). It aligns the PacBio reads in SAMtools and minimap2 ( Li, 2018) and generates coverage tracks for regions of fixed size. In parallel, it queries the GoaT database ( Challis *et al.*, 2023) to identify all matching BUSCO lineages to run BUSCO ( Manni *et al.*, 2021). For the three domain-level BUSCO lineages, the pipeline aligns the BUSCO genes to the UniProt Reference Proteomes database ( Bateman *et al.*, 2023) with DIAMOND ( Buchfink *et al.*, 2021) blastp. The genome is also split into chunks according to the density of the BUSCO genes from the closest taxonomic lineage, and each chunk is aligned to the UniProt Reference Proteomes database with DIAMOND blastx. Genome sequences without a hit are chunked with seqtk and aligned to the NT database with blastn ( Altschul *et al.*, 1990). The blobtools suite combines all these outputs into a blobdir for visualisation.

The genome assembly and evaluation pipelines were developed using nf-core tooling ( Ewels *et al.*, 2020) and MultiQC ( Ewels *et al.*, 2016), relying on the Conda package manager, the Bioconda initiative ( Grüning *et al.*, 2018), the Biocontainers infrastructure ( da Veiga Leprevost *et al.*, 2017), as well as the Docker ( Merkel, 2014) and Singularity ( Kurtzer *et al.*, 2017) containerisation solutions.

Table 4 contains a list of relevant software tool versions and sources.

**Table 4.** Software tools: versions and sources.

Software tool	Version	Source
BEDTools	2.30.0	https://github.com/arq5x/bedtools2
BLAST	2.14.0	ftp://ftp.ncbi.nlm.nih.gov/blast/executables/blast+/
BlobToolKit	4.3.7	https://github.com/blobtoolkit/blobtoolkit
BUSCO	5.4.3 and 5.5.0	https://gitlab.com/ezlab/busco
bwa-mem2	2.2.1	https://github.com/bwa-mem2/bwa-mem2
Cooler	0.8.11	https://github.com/open2c/cooler
DIAMOND	2.1.8	https://github.com/bbuchfink/diamond
fasta_windows	0.2.4	https://github.com/tolkit/fasta_windows
FastK	1.1	https://github.com/thegenemyers/FASTK
Gfastats	1.3.6	https://github.com/vgl-hub/gfastats
GoaT CLI	0.2.5	https://github.com/genomehubs/goat-cli
Hifiasm	0.19.8-r603	https://github.com/chhylp123/hifiasm
HiGlass	1.13.4	https://github.com/higlass/higlass
Merqury.FK	1.1.2	https://github.com/thegenemyers/MERQURY.FK
MitoHiFi	3	https://github.com/marcelauliano/MitoHiFi
MultiQC	1.14, 1.17, and 1.18	https://github.com/MultiQC/MultiQC
NCBI Datasets	15.12.0	https://github.com/ncbi/datasets
Nextflow	23.04.0-5857	https://github.com/nextflow-io/nextflow
PretextView	0.2.5	https://github.com/sanger-tol/PretextView
purge_dups	1.2.5	https://github.com/dfguan/purge_dups
samtools	1.16.1, 1.17, and 1.18	https://github.com/samtools/samtools
sanger-tol/ascc	-	https://github.com/sanger-tol/ascc
Seqtk	1.3	https://github.com/lh3/seqtk
Singularity	3.9.0	https://github.com/sylabs/singularity
TreeVal	1.0.0	https://github.com/sanger-tol/treeval
YaHS	1.2a.2	https://github.com/c-zhou/yahs

### Wellcome Sanger Institute – Legal and Governance

The materials that have contributed to this genome note have been supplied by a Darwin Tree of Life Partner. The submission of materials by a Darwin Tree of Life Partner is subject to the **‘Darwin Tree of Life Project Sampling Code of Practice’**, which can be found in full on the Darwin Tree of Life website here. By agreeing with and signing up to the Sampling Code of Practice, the Darwin Tree of Life Partner agrees they will meet the legal and ethical requirements and standards set out within this document in respect of all samples acquired for, and supplied to, the Darwin Tree of Life Project.

Further, the Wellcome Sanger Institute employs a process whereby due diligence is carried out proportionate to the nature of the materials themselves, and the circumstances under which they have been/are to be collected and provided for use. The purpose of this is to address and mitigate any potential legal and/or ethical implications of receipt and use of the materials as part of the research project, and to ensure that in doing so we align with best practice wherever possible. The overarching areas of consideration are:

•   Ethical review of provenance and sourcing of the material

•   Legality of collection, transfer and use (national and international)

Each transfer of samples is further undertaken according to a Research Collaboration Agreement or Material Transfer Agreement entered into by the Darwin Tree of Life Partner, Genome Research Limited (operating as the Wellcome Sanger Institute), and in some circumstances other Darwin Tree of Life collaborators.

## Data Availability

European Nucleotide Archive: *Vipera berus* (adder). Accession number PRJEB73681; https://identifiers.org/ena.embl/PRJEB73681. The genome sequence is released openly for reuse. The *Vipera berus* genome sequencing initiative is part of the Darwin Tree of Life (DToL) project. All raw sequence data and the assembly have been deposited in INSDC databases. The genome will be annotated using available RNA-Seq data and presented through the Ensembl pipeline at the European Bioinformatics Institute. Raw data and assembly accession identifiers are reported in Table 1 and Table 2.

## Author information

Members of the Wellcome Sanger Institute Tree of Life Management, Samples and Laboratory team are listed here: https://doi.org/10.5281/zenodo.12162482.

Members of Wellcome Sanger Institute Scientific Operations: Sequencing Operations are listed here: https://doi.org/10.5281/zenodo.12165051.

Members of the Wellcome Sanger Institute Tree of Life Core Informatics team are listed here: https://doi.org/10.5281/zenodo.12160324.

Members of the Tree of Life Core Informatics collective are listed here: https://doi.org/10.5281/zenodo.12205391.

Members of the Darwin Tree of Life Consortium are listed here: https://doi.org/10.5281/zenodo.4783558.
