# Supplementary material for: The genome sequence of the common adder, Vipera berus (Linnaeus, 1758)
Source: Wellcome Open Res. 2025 Sep 26;10:11. Originally published 2025 Jan 14. [Version 2] doi: 10.12688/wellcomeopenres.23470.2 (PMC12511858; doi:10.12688/wellcomeopenres.23470.2)
Supplement: Peer review history

# Reviewer report: Reviewer response for version 2

**DOI:** 10.21956/wellcomeopenres.27561.r134749

**Published:** 2025-10-09

**Authors:** Takushi Kishida

**Affiliations:** 1 Nihon University, Kanagawa, Japan

This study provides a phased genome assembly of a common adder. Quality of the genome assembly is excellent, and I have no comments on the methodologies. I have only one very minor comment:

 Background section: “It ranges from the United Kingdom, where it is the only venomous snake species, …”; It might be better to replace the term “United Kingdom” with “Great Britain” because this species is not distributed in the Island of Ireland.

Are sufficient details of methods and materials provided to allow replication by others?

Yes

Is the rationale for creating the dataset(s) clearly described?

Yes

Are the datasets clearly presented in a useable and accessible format?

Yes

Are the protocols appropriate and is the work technically sound?

Yes

Reviewer Expertise:

evolutionary genomics

I confirm that I have read this submission and believe that I have an appropriate level of expertise to confirm that it is of an acceptable scientific standard.

# Reviewer report: Reviewer response for version 1

**DOI:** 10.21956/wellcomeopenres.25881.r121687

**Published:** 2025-04-10

**Authors:** Jia-Tang Li

**Affiliations:** 1 University of Chinese Academy of Sciences, Beijing, People's Republic of China, China

The manuscript entitled ‘The genome sequence of the common adder, *Vipera berus* (Linnaeus, 1758)’ presented a chromosome-level genome assembly from an individual female *Vipera berus*, containing two haplotypes. The genome data was an important addition to viper population genetics, biogeography, and venom research, making this manuscript and data significant and merit for publication. My only suggestion on the manuscript is that it would be better to add some comparisons with the quality of genome assembly in closely related species. Also, please make annotation files based on transcriptome data publicly available in a timely manner.

Are sufficient details of methods and materials provided to allow replication by others?

Yes

Is the rationale for creating the dataset(s) clearly described?

Yes

Are the datasets clearly presented in a useable and accessible format?

Yes

Are the protocols appropriate and is the work technically sound?

Yes

Reviewer Expertise:

Evolutionary Biology

I confirm that I have read this submission and believe that I have an appropriate level of expertise to confirm that it is of an acceptable scientific standard.

# Reviewer report: Reviewer response for version 1

**DOI:** 10.21956/wellcomeopenres.25881.r118829

**Published:** 2025-03-07

**Authors:** Hans Recknagel

**Affiliations:** 1 University of Ljubljana, Ljubljana, Slovenia

The authors provide a full genome assembly of the European viper ( *Vipera berus*), a venomous snake species widely distributed across Eurasia. The species is of ecological and evolutionary interest, including its widespread colour polymorphism. Using a combination of Pacbio HiFi and Hi-C sequencing, the authors construct a chromosome-scale assembly. The genome provides a very valuable resource representing one of the most widespread and cold-adapted reptiles worldwide.

 The article is succinct and well-written. I have no major issues. In the following, I ask the authors to clarify a few points or expand on their explanations:

1. Replace term “ovoviviparous” with “viviparous” (see reasoning for example here: Blackburn, D. G. 2000. Classification of the reproductive patterns of amniotes. *Herpetological Monographs*, 371-377.)
2. The second and third paragraph could be complemented with data across its distribution and not limited to the UK to give a better perspective if possible. For example, in the second paragraph it would be interesting to not only mention adders biting humans but also dogs (and cattle?), (e.g. “Canine adder bites in the UK: a retrospective study of cases reported to the Veterinary Poisons Information Service”, “Clinical and biochemical changes in 53 Swedish dogs bitten by the European adder – *Vipera berus*.”).
3. Figure 2: It seems that the GC content is not showing any variation across the genome in this Figure, but in others tables variation (albeit small) across chromosomes is present. Is this due to the low resolution of the y-scale of the GC content in the Figure? I assume this is the case, as the smallest scaffolds seem to show some variation as visible in the section between 98-100%.
4. Figure 3: The colours for no-hit and for Chordata are very similar. Especially since overlapping circles are semi-transparent, the mixing of colours makes it hard to see what is darker due to overlap and what due to being Chordate. My understanding is that the vast majority of the scaffolds and especially the large ones are chordate hits, but it would be good to just have a more distinct colour for no-hits.
5. Figure 5: It would be useful to have Z and W chromosomes directly marked in the Figure, for example by including a coloured or thicker outline for those Hi-C scaffolds.
6. The methods section states that a single blood sample was taken. All laboratory analyses were done on this single sample, which is impressive. It would be great if the total volume of blood extracted could be included in case it was measured. Also for each of the DNA/RNA extraction steps it would be great to know the starting material amount.

Are sufficient details of methods and materials provided to allow replication by others?

Yes

Is the rationale for creating the dataset(s) clearly described?

Yes

Are the datasets clearly presented in a useable and accessible format?

Partly

Are the protocols appropriate and is the work technically sound?

Yes

Reviewer Expertise:

Evolutionary Biology, Herpetology, Phylogenetics, Genomics

I confirm that I have read this submission and believe that I have an appropriate level of expertise to confirm that it is of an acceptable scientific standard, however I have significant reservations, as outlined above.

## References

[1] Classification of the reproductive patterns of amniotes. Herpetological Monographs, Blackburn, D. G.2000.371-377;

# Response

**Published:** 2025-09-22

**Authors:** Tree of Life Team Sanger

**Affiliations:** Wellcome Trust Sanger Institute, UK

Thank you for reviewing this data note. We have submitted a new version of the article, and our responses to your specific comments are given below.

 1. Replace term “ovoviviparous” with “viviparous” (see reasoning for example here: Blackburn, D. G. 2000. Classification of the reproductive patterns of amniotes. *Herpetological Monographs*, 371-377.)

***Response: **We have made this change.* 

 2. The second and third paragraph could be complemented with data across its distribution and not limited to the UK to give a better perspective if possible. For example, in the second paragraph it would be interesting to not only mention adders biting humans but also dogs (and cattle?), (e.g. “Canine adder bites in the UK: a retrospective study of cases reported to the Veterinary Poisons Information Service”, “Clinical and biochemical changes in 53 Swedish dogs bitten by the European adder – *Vipera berus*.”).

***Response:** We appreciate the suggestion. We have opted to retain the current text, which briefly illustrates human envenomation in the UK as a familiar context for readers. Additional examples of bites in other animals are available in the literature but were not included here to keep the introduction concise.*

 3. Figure 2: It seems that the GC content is not showing any variation across the genome in this Figure, but in other tables variation (albeit small) across chromosomes is present. Is this due to the low resolution of the y-scale of the GC content in the Figure? I assume this is the case, as the smallest scaffolds seem to show some variation as visible in the section between 98-100%.

***Response: **In Figure 2 where the GC track reflects GC proportion across bins ordered by scaffold size. The largest scaffolds, which comprise the bulk of the assembly, have very similar GC content, so the track appears flat. Small scaffolds at the right-hand end of the plot (~98–100%) show more variation, but these contribute little to the overall pattern.*  

 4. Figure 3: The colours for no-hit and for Chordata are very similar. Especially since overlapping circles are semi-transparent, the mixing of colours makes it hard to see what is darker due to overlap and what due to being Chordate. My understanding is that the vast majority of the scaffolds and especially the large ones are chordate hits, but it would be good to just have a more distinct colour for no-hits.

***Response: **BlobToolKit is designed to be interactive and the palette can be altered for different phyla. We have changed the static figure in this data note to a light orange for the no-hit contigs.*

 5. Figure 5: It would be useful to have Z and W chromosomes directly marked in the Figure, for example by including a coloured or thicker outline for those Hi-C scaffolds.

***Response:** We have included a labelled PretextView map as the static view, so that the Z and W chromosomes are easy to see.*

 6. The methods section states that a single blood sample was taken. All laboratory analyses were done on this single sample, which is impressive. It would be great if the total volume of blood extracted could be included in case it was measured. Also for each of the DNA/RNA extraction steps it would be great to know the starting material amount.

***Response: **We do not have records of the exact blood volume or input amounts for each extraction step, so we are unable to provide these details.*
